# An Interesting Case of Wolf-Parkinson-White Syndrome in a Young Patient With Sensorineural Deafness

**DOI:** 10.7759/cureus.62928

**Published:** 2024-06-22

**Authors:** Zahid Khan, Ayub Khan

**Affiliations:** 1 Acute Medicine, Mid and South Essex NHS Foundation Trust, Southend on Sea, GBR; 2 Cardiology, Bart’s Heart Centre, London, GBR; 3 Cardiology and General Medicine, Barking, Havering and Redbridge University Hospitals NHS Trust, London, GBR; 4 Cardiology, Royal Free Hospital, London, GBR; 5 Emergency Medicine, Barking, Havering and Redbridge University Hospitals NHS Trust, London, GBR

**Keywords:** stroke-like episodes (melas), av reentrant tachycardia (avrt), hypertrophic obstructive cardiomyopathy (hocm), left ventricular outflow tract obstruction (lvot), accessory atrioventricular pathways, radiofrequency catheter ablation (rfca), delta wave, leopard syndrome, sensorineural deafness, wolf-parkinson-white syndrome

## Abstract

Wolff-Parkinson-White (WPW) syndrome is a condition associated with tachycardia due to accessory pathways in the heart, and it is one of the most common causes of tachycardia in infants and children. WPW may also be associated with mitochondrial encephalomyopathy, lactic acidosis, stroke-like episodes (MELAS syndrome) or LEOPARD syndrome (LS). We report a case of pre-excitation WPW syndrome in a 17-year-old man who was brought to the hospital by ambulance following the collapse. WPW syndrome type A was diagnosed from precordial leads. Electrocardiography (ECG) revealed a short PR interval, delta waves, and positive waves with dominant R in all pericardial leads. Blood test results showed an isolated elevated ALT level. Subsequent echocardiography was unremarkable, with an ejection fraction of 55%, apart from septal and inferior wall dyssynchrony. With regard to the past medical history, he had sensorineural deafness (SND) since childhood and had a family history of SND. Consequently, the patient was transferred to the cardiac electrophysiology department at another hospital after consultation and underwent ablation. A successful post-ablation electrocardiogram revealed the resolution of the WPW syndrome signs and post-ablation features, such as peak T waves.

## Introduction

Patients with Wolff-Parkinson-White (WPW) syndrome have accessory atrioventricular pathways (AP) that connect the atrium to the ventricle outside the normal atrioventricular (AV) nodal conduction system. The annual incidence of this disorder ranges between 0.1% and 0.3% in the general population and is associated with a sudden cardiac death (SCD) risk of less than 0.6%. The atria and ventricles in a normal heart are electrically isolated from each other by a non-conductive fibrous AV ring, except at the AV node and bundle of His. The electrical impulse is generally initiated within the sinoatrial node and is conducted to the ventricles via the His-Purkinje system. Patients with WPW have at least one additional accessory electrical pathway that bypasses the AV node, resulting in ventricular pre-excitation by producing premature electrical impulses. This, in turn, allows the retrograde propagation of impulses. The accessory pathway can be associated with re-entrant SVT and sudden death, depending on conduction characteristics. The syndrome is further categorized as type A or B based on conventional 12-lead electrocardiography (ECG). Patients with type A WPW syndrome have pre-excitation from the posterolateral base of the left ventricle (LV) and the interventricular septum or right ventricular free wall in WPW type B [1]. Patients with WPW may experience various symptoms, such as dizziness, palpitations, congestive heart failure, syncope, and SCD. The key ECG features of WPW include a short PR interval of <0.12 seconds, a widened QRS complex with a total duration of >0.12 seconds, slurring and the slow rise of the initial QRS complex (delta wave), and abnormal ventricular repolarization. AP results in abnormal pre-excitation around the AV annuli in patients with WPW, leading to dyssynchronous contraction of heart chambers [2]. Patients who undergo radiofrequency catheter ablation (RFCA), which is a well-established treatment for WPW syndrome, have a tachyarrhythmia recurrence risk of less than 5%. Successful ablation of the accessory pathway depends on the precise localization of the pathway [3].

## Case presentation

A 17-year-old male patient was brought to the emergency department by ambulance service following a collapse at home. He was walking to the kitchen when he collapsed, with a transient loss of consciousness. The episode was witnessed by his mother, who heard a bang in the kitchen. She saw the patient lying on the floor who regained consciousness quickly and there was no evidence of any jerky body movements, tongue biting, or incontinence. He denied having any head injury due to a fall. He regained consciousness after two minutes. Furthermore, he had palpitations over the two weeks prior to this episode of collapse. He was studying in college and denied any strenuous physical activity. In terms of past medical history, he had sensorineural deafness (SND) since childhood. He also had two first-degree relatives with SND; however, there was no family history of SCD. He denied the use of any recreational drugs or alcohol and was not taking any regular medications.

**Figure 1:**
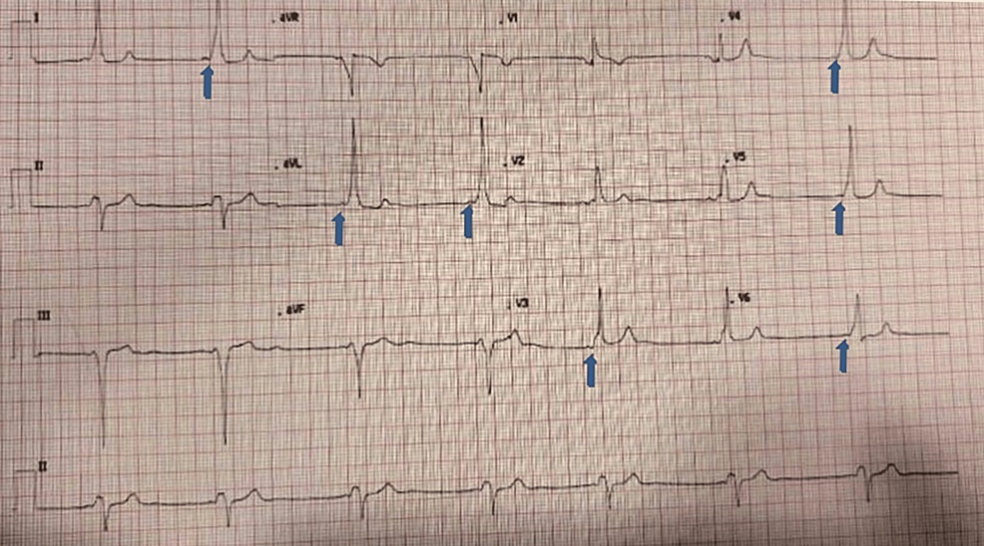
Electrocardiogram showed a short PR interval, normal QRS complexes and delta waves consistent, left axis deviation, prominent T waves in inferior leads, and V3-V6 precordial leads (blue arrows pointing to delta waves)

**Figure 2:**
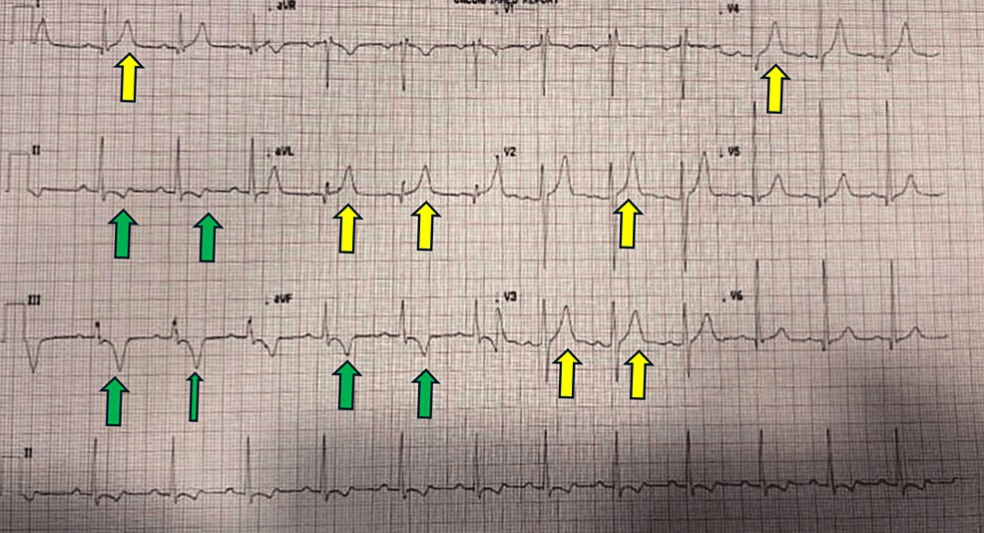
Electrocardiogram post-ablation showing normal sinus rhythm with deep inverted T waves inferiorly (Green arrows), right bundle branch block, and tall T waves anterolaterally (Yellow arrows)

His clinical examination was unremarkable, and there was no evidence of physical injury from a fall. His cardiovascular examination was unremarkable, and the Glasgow Coma Scale score was 15. The patient did not exhibit any focal neurological deficits. His initial electrocardiogram showed a short PR interval, normal QRS complexes and delta waves consistent, S waves in lead V1 and left axis deviation, prominent T waves in inferior leads, and V3-V6 precordial leads. These findings are consistent with type B Wolff-Parkinson-White (WPW) syndrome (Figure 1). He also had a normal chest radiograph, and routine blood tests were normal, apart from elevated ALT levels. Echocardiography revealed normal biventricular function with no valvular abnormalities. The patient was discussed with the tertiary cardiac electrophysiology team and transferred for radiofrequency catheter ablation (RFCA) of the accessory pathway. The patient underwent electrophysiological studies, and successful accessory pathway ablation was performed. ECG post-ablation is shown in Figure 2. He was followed up in the outpatient department for three months and remained symptom-free.

## Discussion

Differential diagnosis

The differential diagnosis of WPW includes structural heart disease, other arrhythmias, and hypertrophic obstructive cardiomyopathy (HOCM). Patients with HOCM may also present with collapse and loss of consciousness. Patients with HOCM usually have a hypertrophied left ventricular (LV) septum in the basal LV segment, and they may also have left ventricular outflow tract obstruction (LVOT), diastolic dysfunction, mitral regurgitation, and left atrial enlargement if they have diastolic dysfunction. The patient had a normal echocardiogram, ruling out HOCM. Other differential diagnoses can be broadly divided into narrow, complex, regular, and irregular tachycardia. Narrow-regular narrow complex tachycardia includes sinus or atrial tachycardia, atrial flutter, junctional tachycardia, atrioventricular re-entrant tachycardia (AVRT), and atrioventricular nodal re-entrant tachycardia (AVNRT). The ECG and electrophysiological studies supported the diagnosis of WPW in this patient. Irregular narrow complex tachycardia includes sinus tachycardia with ectopic beats, atrial flutter (with variable AV block), atrial fibrillation, and multifocal atrial tachycardia. Regular wide complex tachycardia includes ventricular tachycardia; paced rhythm; accelerated idioventricular rhythm; artefacts; accessory pathways that can be triggered by drug, metabolic, and electrolyte abnormalities; and SVT associated with aberrant ventricular conduction. Finally, irregular wide complex tachycardia includes torsade de pointes and nonsustained ventricular tachycardia, and any cause of irregular narrow complex tachycardia is associated with abnormally aberrant conduction. Brugada syndrome is another possible differential diagnosis in young patients who present with collapse [4]. After confirmation of the diagnosis and discussion with the electrophysiology department, the patient underwent successful radiofrequency ablation for the WPW syndrome.

Discussion

**Figure 3:**
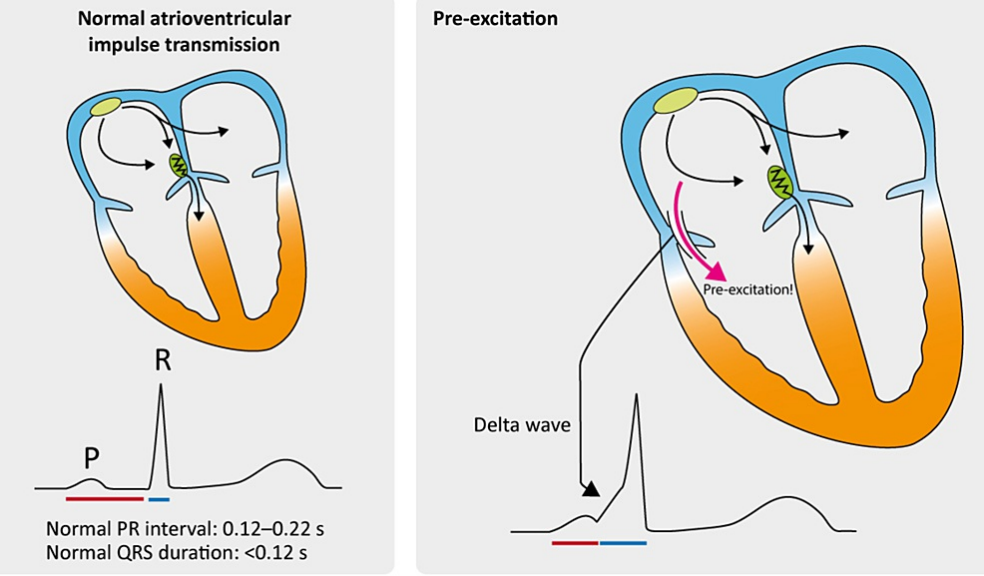
Electrocardiographic findings during normal AV conduction and during pre-excitation Permission obtained from the author for image reproduction [8]. AV, atrioventricular

**Figure 4:**
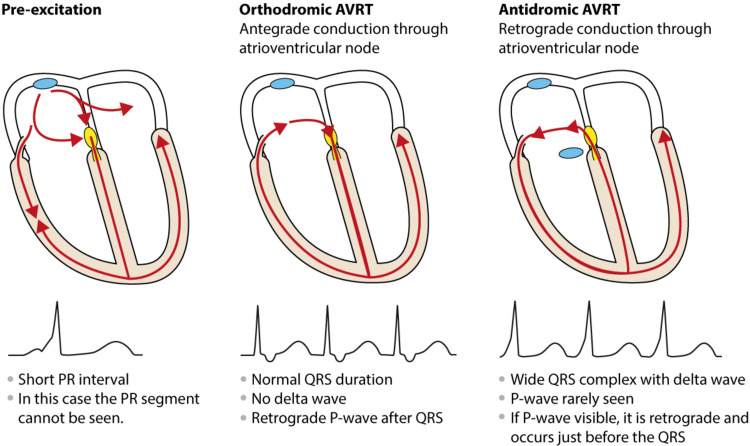
Electrocardiographic features of pre-excitation, orthrodromic, and antidromic AVRT Permission obtained from the author for reproducing the figure [8]. AVRT, antiventricular re-entrant tachycardia

The prevalence of WPW syndrome is two per 1000 general population [4,5]. Tachyarrhythmia in the presence of an accessory pathway may present with distinct electrocardiographic features, which can be misdiagnosed and treated, resulting in life-threatening complications. Patients with WPW have fast anteretrograde conduction through the bundle of Kents, which is also known as an accessory pathway, which can outpace slower AV node conduction. Quick ventricular depolarization as a result of this conduction results in distinct ECG changes, such as a virtually pathognomonic delta wave, wide QRS complex, and short PR interval. It can be difficult to detect electrocardiographic anomalies at baseline in patients with WPW because the accessory pathway may not be conducted in an anterograde manner. Most patients with WPW develop paroxysmal AVRT via anterograde conduction through the AV node, followed by retrograde conduction through the bundle of Kent (orthodromic AVRT), which produces narrow complex tachycardia [6,7]. Patients with WPW fibrillation may not demonstrate typical rapid pre-excitation responses. This is likely due to the anterograde conduction delay of the accessory pathway relative to the AV node or AV blocks (Figures 3, 4).

Patients with antidromic AVRT tend to have wide QRS complex tachycardia because anterograde conduction through the accessory pathway is followed by retrograde conduction through the AV node. Similar circuits may also be present in patients with pre-existing bundle branch blocks [5-8]. The atria can discharge at a rate of >300 impulses per minute, which can obscure the delta waves commonly observed in patients with WPW syndrome. The AV node has an intrinsic repolarization property allowing the node to slow down faster signals and block most of these impulses due to decremental conduction. On the other hand, patients with an accessory pathway lack this protection, making 1:1 conduction possible, resulting in increased ventricular rates reaching 300 beats per minute (bpm). Patients with pre-excited AF are hence characterized by malignant arrhythmia, and the rhythm can quickly degenerate into ventricular fibrillation (VF), resulting in sudden death [6,7]. Patients who undergo accessory pathway ablation can develop large peaked T waves in the same leads along with the delta wave, which was also notable, with concordant polarity. This is more commonly seen in delta and T waves positive leads (leads I, aVL, and V2-6 in our patient).

Certain medications such as beta-blockers and calcium channel blockers such as Diltiazem, Verapamil, Digoxin, and adenosine should be avoided in patients with WPW, as they can accelerate the accessory pathway. Additionally, young patients with WPW may not show a typical short PR interval or delta waves, and young patients presenting with collapse should be properly investigated. Patients with WPW tachycardia may have underlying infection, pulmonary embolism, or dehydration. Multiple accessory pathways are possible in approximately 10% of the cases. The rate of SCD ranges from 0.0002 to 0.0015 per patient-year in patients with WPW [5,6]. Certain high-risk factors for SCD in patients with WPW include male sex, history of atrial fibrillation or AVRT, multiple accessory pathways, age less than 35 years, and septal location of the accessory pathway. The overall prognosis of patients with WPW has significantly improved because of its dangerous nature due to advanced antiarrhythmic medications and ablation techniques. A classic post-ablation memory T-wave pattern often provides evidence of successful ablation. RFCA has a higher success rate and lower rate of complications and can be curative in these patients [9,10].

Another interesting fact about our patient was his strong family history of SND, which he also experienced. However, we were unable to establish any link between the two. Sung et al. reported the presence of SND in a patient with WPW who was also diagnosed with mitochondrial encephalopathy, lactic acidosis, and stroke-like episodes (MELAS) syndrome who did not complain of any deafness but was found to have SND on pure-tone audiometry [11]. Wolff-Parkinson-White (WPW) syndrome should always be considered as a possible differential diagnosis in a young patient presenting with collapses or symptomatic palpitations. Seeking specialist advice is recommended for patients with complex tachyarrhythmia who need further evaluation and treatment. Young patients with WPW syndrome can die due to tachyarrhythmia and VF if misdiagnosed, particularly athletes [10].

## Conclusions

Patients with WPW syndrome are at high risk of SCD due to the development of VF from the accessory pathway. This can be exacerbated by AV nodal-blocking medications in patients with pre-excited atrial fibrillation and flutter. Patients with WPW should undergo electrophysiological testing for risk stratification. Catheter ablation is the first-line therapy for patients with a history of arrhythmia and pre-excitation on ECG.

## References

[1] Tanabe J, Watanabe N, Yamaguchi K, Tanabe K (2021). A case of Wolff-Parkinson-White syndrome in which two-dimensional speckle-tracking echocardiography was useful for identifying the location of the accessory atrioventricular pathway. Eur Heart J Case Rep.

[2] Cai Q, Shuraih M, Nagueh SF (2012). The use of echocardiography in Wolff-Parkinson-White syndrome. Int J Cardiovasc Imaging.

[3] Jackman WM, Wang XZ, Friday KJ (1991). Catheter ablation of accessory atrioventricular pathways (Wolff-Parkinson-White syndrome) by radiofrequency current. N Engl J Med.

[4] Wilde AA, Antzelevitch C, Borggrefe M (2002). Proposed diagnostic criteria for the Brugada syndrome: consensus report. Circulation.

[5] Klein GJ, Gula LJ, Krahn AD, Skanes AC, Yee R (2009). WPW pattern in the asymptomatic individual: has anything changed?. Circ Arrhythm Electrophysiol.

[6] Uemura T, Kondo H, Shinohara T (2023). Multiple accessory pathways coexisting with a persistent left superior vena cava: a case report. J Med Case Rep.

[7] Jung HJ, Ju HY, Hyun MC, Lee SB, Kim YH (2011). Wolff-Parkinson-White syndrome in young people, from childhood to young adulthood: relationships between age and clinical and electrophysiological findings. Korean J Pediatr.

[8] (2024). Arrhythmia and arrhythmology. https://ecgwaves.com/lesson/arrhythmias-and-arrhythmology/.

[9] Masukume G, Dixon M (2022). Wolff-Parkinson-White syndrome type B. Pan Afr Med J.

[10] Kulig J, Koplan BA (2010). Wolff-Parkinson-White syndrome and accessory pathways. Circulation.

[11] Sung JH, Han JH, Kim H, Kim JB (2018). Wolff-Parkinson-White syndrome in a patient with mitochondrial encephalopathy, lactic acidosis, and stroke-like episodes syndrome mimicking juvenile myoclonic epilepsy. J Clin Neurol.

